# Developed steady-state response for a new hybrid damper mounted on structures

**DOI:** 10.1371/journal.pone.0290248

**Published:** 2023-08-17

**Authors:** Mohammad Ziaee, Farzad Hejazi

**Affiliations:** 1 Department of Civil Engineering, University Putra Malaysia, Seri Kembangan, Malaysia; 2 Faculty of Environment and Technology, The University of The West England, Bristol, United Kingdom; National University of Sciences and Technology, PAKISTAN

## Abstract

Coulomb friction is considered as a mechanical approach to diminish the structural responses during the excitations. However, in case of severe oscillations supplementary mechanisms are employed besides the friction to mitigate the destructive effects of the vibrations in structures. Therefore, the main goal of this research is to develop a new Hybrid System (HS) which is a parallel combination of Viscous Damping (VD) and Coulomb friction for structures subjected to dynamic load. To achieve this goal, the effect of viscous damper is embedded in the equation of motion which is proposed by Den Hartog for a Single-Degree-of-Freedom (SDOF) Coulomb system, and has been extensively implemented for past few decades. In the considered numerical example in this study, implementing the proposed HDM in system resulted in decreasing the maximum displacement in the range of 1% to 98% for different amounts of force amplitude and viscous damping ratios. Also, applying the proposed HDM increased the time lag for about up to 24% for the frequency ratios greater than 1. The developed hybridized system in this study can be utilised as new generation of Tuned Mass Damper (TMD) to improve their energy dissipating efficiency under severe excitations.

## Introduction

Various attempts have been made to understand the friction mechanism of sliding objects yet, most of them were unsuccessful [1]. It is possible to encounter the friction force as an energy dissipator in any type of industrial machinery or civil structure where the relative motion of parts comes to appear [2, 3]. In structures, the energy dissipation can occur through structural material or the component friction which is classified as the inherent damping. However, in the case of severe excitations, the inherent damping is not capable to reduce the structural response to an acceptable range. Therefore, there is a need to utilise supplementary damping besides the inherent damping to overcome the shortcomings. Supplementary dampers are artificial energy dissipators that increase the total structural damping, reduce the structural response to external vibrations and contribute to structure resistance against severe translations. Various damping mechanisms such as the viscous dampers [4], yielding dampers, magnetic dampers, tuned mass dampers, friction dampers [5] and tuned liquid dampers [6–8] are frequently applied in the newly built structures.

In addition, base isolators are recently introduced techniques to separate the main structure from the base and reduce the negative effect of base motion on the superstructure [9–11]. Whatever the choice of damping system is, the friction performs the main role in diminishing the structural responses through the energy dissipation and therefore, its effect on the dynamic systems is required to be studied carefully.

The Coulomb system is a conventional mechanical model in which the mass is connected to support by the aid of a massless spring and the kinetic friction is applied to the mass while it is moving on a rough surface. The principal issue in oscillatory movement in systems with friction is to avoid getting into the sticking phase. Thus, to overcome this problem, it is crucial to extract a new steady-state solution of the oscillatory system and set the force amplitude ratio such that the system has a zero-duration sticking phase. However, due to the non-linearity of the equation of the motion and bilateral behaviour of the Coulomb friction force finding the steady-state response of the system is somehow complicated. In 1931, Den Hartog [12] did the early research to find a response for the Coulomb systems. By taking some assumptions finally he succeeded to obtain a useful steady-state response for SDOF vibratory system under external harmonic load and constant frictional force.

The simplicity of his proposed method is due to not only its basic assumptions which are good approximations of what happens in the reality but also its simple calculation steps. In the study conducted by Den Hartog, the formulation of Maximum Displacement (MD) was derived and the design procedure was continued with the aid of static methods, which are popular amongst civil engineers.

In addition, complementary studies have been carried out to find the response of the dynamic systems by applying a variety kind of mathematical techniques, for instance, the time-domain numerical integration methods [13] and the phase plane method [12, 14, 15] were used in several types of research to investigate the behaviour of the dynamic models equipped with energy dissipators and supplementary dampers. Also in this regard, H.-K Hong et al. in 2000 [5], tried to calculate the Maximum Velocity (MV) beside the Maximum Displacement (MD) in a Coulomb system subjected to the lateral loading. It is noticeable that H.-K Hong et al. also proposed a straight solution for the steady-state response of the SDOF systems in presence of the Coulomb friction [16], however, because of its complicated nature it is not frequently used by the structural designers. Then, in another recent research performed by D. J. Riddoch et al. in 2020 [17], the structural parameters of an SDOF oscillatory system subjected to friction and the base motion was found based on Den Hartog’s formulations [12]. Furthermore, in another research, the multi-mass system in the presence of the friction and subjected harmonic loads has been interrogated by L. Marino et al. (2021) [18] and its steady-state response has been calculated as well. Finally, M. Ziaee et al. (2022) carried out a study to propose a new steady-state solution for hybrid systems [19], however, the method is replaced by simpler calculations in the present research and can be handled more easily. Also, the present research amends the Den Hartog formulation as in real structures always there is some inherent damping which is ignored by Den Hartog’s method.

As discussed above, prior investigations were about the Coulomb systems and the necessity to find an acceptable steady-state response for them. However, in the present work, the main focus is on developing Den Hartog’s method into a dynamic hybridized system with the presence of both Coulomb friction and viscous damper. As the previously investigated dampening systems were ineffective to mitigate the response of the structures under lateral loading, throughout this research the structural designers would be able to seize the opportunity to design the structures equipped with two energy dissipation mechanisms [20] which can ameliorate their performance under severe excitations. Also, the proposed system in this work can be compared to TMDs as it shows the same behaviour and can be utilised as new generation of TMD in real structures in comparison to other carried out studies [21, 22]. TMDs and their effectiveness are widely investigated by researchers [23, 24] as in the case of severe excitations they can be optimized to suppress the structural vibrations [25–27]. Conversely, the HDM demonstrates more resistant against mistuning in comparison with conventional TMDs [28].

Therefore, Den Hartog’s method is expanded to this proposed hybridized dynamic system as it contains less calculation steps. By embedding the effect of the Viscous Damper (VD) in the main equation of the motion, MD and its corresponding time lag can be found easily. Besides finding the system parameters additional effort is done to calculate the the steady-state response of the developed hybrid system. And finally, to avoid the sticking phase in the calculations a boundary limit for *α* is determined which can be considered as a handy design criteria for the structural designers.

### 1. Developing a new hybrid damping mechanism

Nowadays, structures are frequently subjected to dynamic loads which in the long term will have harmful effect on the structure’s stability and highly reduce operation life of structures. Consequently, in order to protect structure against applied excitation, it is necessary to dissipate vibration effect on structure. Therefore, the inherent damping performs a key role in exhausting the excessive energy of structures caused by the external loads. However, in case of severe excitations, the effectiveness of inherent damping does not seem enough to diminish structure oscillations and therefore there is a need to ameliorate the efficiency of vibration control using the supplementary damping system such as dampers.

Therefore, as a solution, in this research an HDM is developed by implementing and combining the supplementary dampers and Coulomb friction in structure. The proposed HDM is capable to increase the amount of energy dissipation and consequently reduces the structural response significantly.

Therefore Single-Degree-of-Freedom (SDOF) System is considered due to its simplicity to prove the efficiency of the proposed HDM for structure and development of formulations regarding structural response under applied excitations.

**Fig 1:**
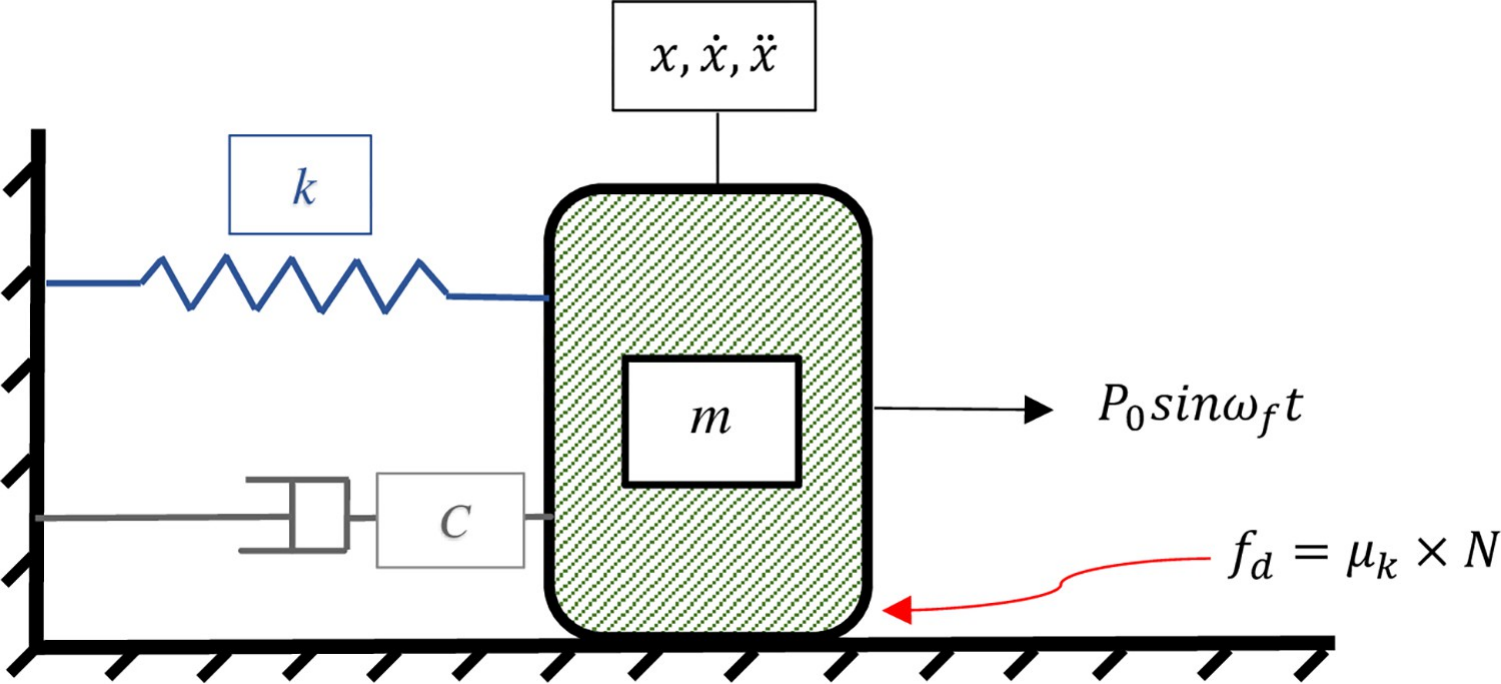
Developed hybrid damping mechanism in schematic view.

To commence the formulation of the SDOF system’s parameters, the schematic model of the proposed hybrid system is sketched up as shown in Fig 1.

In where, *P*_0_ and *ω*_*f*_ denote the amplitude and angular frequency of the externally applied load respectively and *t* points to elapsed time. Other parameters are *m*, *c* and *k* where the first one represents the mass of the system, the second one shows the damping coefficient and the last one is the stiffness of the mass-less spring. Also, by assuming that the SDOF structure is oscillating on a horizontal surface, the vertical load component applied to the hybrid system and perpendicular to the direction of the motion (*N*) is equal to the product of mass by the gravity acceleration. Multiplying the *N* by kinetic friction coefficient *μ*_*k*_ results in *f*_*d*_ which is known as the kinetic friction force or Coulomb friction. In this case, the governor equation of the motion for the proposed SDOF system with the Hybrid Damping Mechanism (HDM) can be formed as:

$$m\ddot{x}+c\dot{x}+kx=P_{0}sin\omega_{f}t-f_{d}$$ (1)

**Fig 2:**
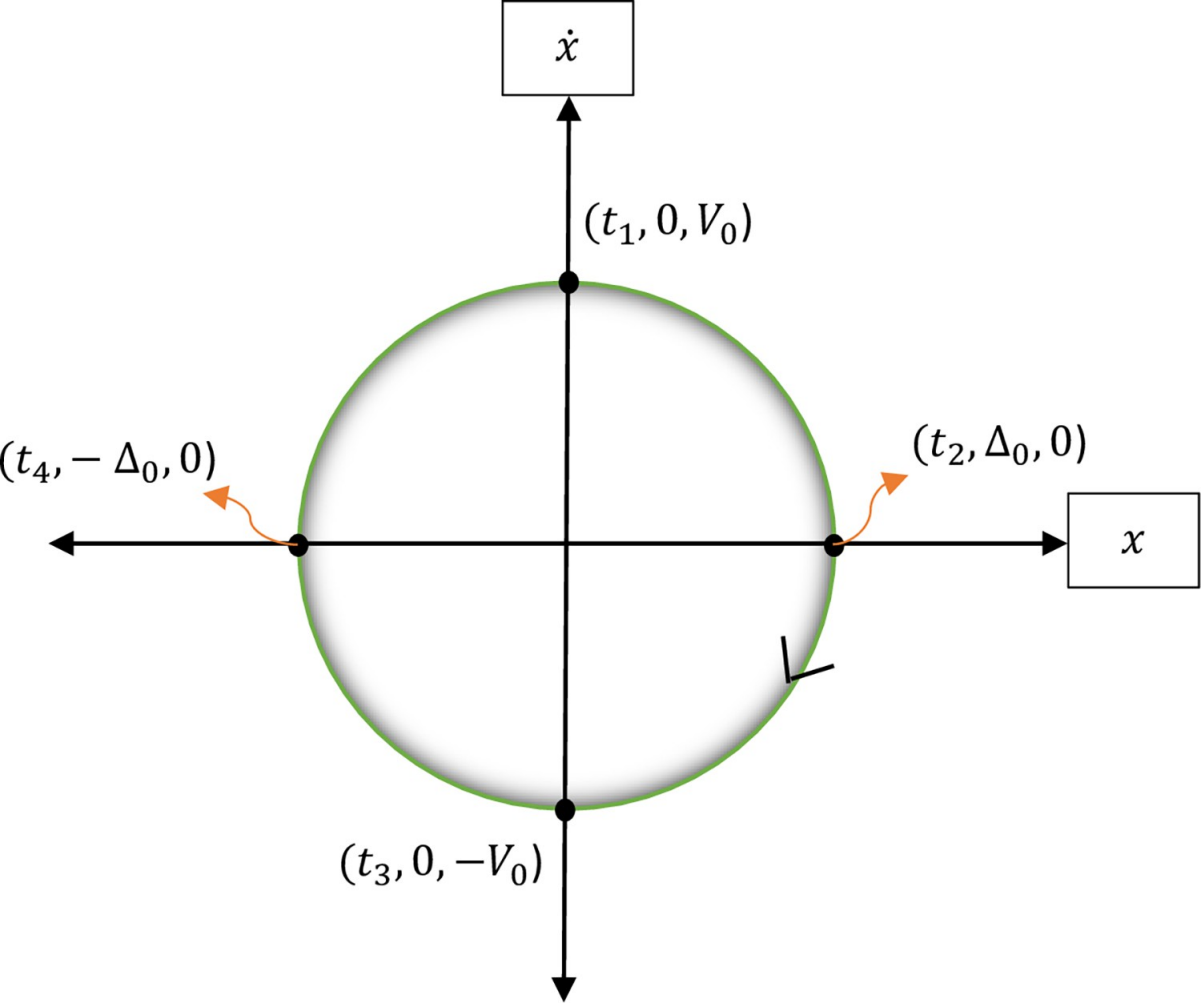
The schematic cycle for non-sticking motion.

In which $\dot{x}$ and $\ddot{x}$ designate velocity and acceleration which are the first and second derivatives of displacement with respect to time respectively. Then to formulate the steady-state response of the proposed SDOF system a zero-duration sticking cycle of motion is considered and it is presumed that the phase plane curve ($x,\;\dot{x}$) has a symmetrical form as illustrated in Fig 2. Therefore, it seems sufficient to consider only one half of the curve, for instance, the lower branch which is similar to Den Hartog’s assumption [2].

To begin the formulation, the steady-state solution of the equation of motion is supposed to be similar to one presented by Den Hartog in 1931 [2]. However, to develop the results for the proposed hybrid system the effect of VD is embedded in the principal equation of the motion of the SDOF system

So, after the differential equation of the motion is solved for the proposed SDOF hybrid system and the steady-state response is found. The new form of steady-state response passes throughout four specific points and unlike the H.-K Hong et al.’s method it is hypothesized that the maximum velocity (MV) occurs on the $\dot{x}$ axis and so there is not any deviation and time lag attributed to it.

According to Fig 2, the displacement and velocity for different time steps are as:

$$(0,+V_{0})@t_{1},\;{(\Delta }_{0},0)@t_{2},\;(0,-V_{0})@t_{3},\;{(-\Delta }_{0},0)@t_{4}$$ (2)

In which the time steps are defined using the below formula:

$$t_{4}=t_{2}+\pi /\omega_{f}$$ (3)

By applying the boundary conditions to the derived steady-state solution the finalised form of the steady-state response of the hybridized SDOF system can be obtained. This derived solution is similar to the result achieved by Den Hartog in 1931 [2]. However, there are some differences between Den Hartog’s solution and the proposed solution in this research which are attributed to the presence of the viscous damper in the SDOF system.

The developed damping mechanism can be also utilised in the structures as Tuned Mass Dampers (TMD) and capable to resolve the main drawback of TMDs which is the large amplitude of the motion for the tuned auxiliary mass.

### 2. Shaping the equation of motion of the hybrid system for the lower branch

For this purpose, by selecting the lower path of Fig 2 for the time step *t*_2_≤*t*≤*t*_4_, the steady-state formula of the hybrid SDOF system in the clockwise direction takes the form of:

$$x(t)=Acos(\omega_{f}t+\phi )+B+e^{-\xi \omega_{n}t}(asin\omega_{d}t+bcos\omega_{d}t)$$ (4)

In which;

$$A=\frac{P_{0}}{k\sqrt{{\left(1-\beta^{2}\right)}^{2}+{\left(2\xi \beta \right)}^{2}}},\;B=\frac{A\sqrt{{\left(1-\beta^{2}\right)}^{2}+{\left(2\xi \beta \right)}^{2}}}{\alpha },\;\alpha =\frac{P_{0}}{{+f}_{d}}$$ (5)

$$\beta =\frac{\omega_{f}}{\omega_{n}},\;\omega_{n}=\sqrt{\frac{k}{m}},\;\omega_{d}=\omega_{n}\sqrt{1-\xi^{2}},\;\xi =2m\omega_{n}$$ (6)

Where *B* can be expressed as static displacement of the friction force, *β* is the frequency ratio and *ω*_*n*_ is the frequency of the main structure, *ω*_*d*_ is the frequency of the HDS, *ξ* represents the damping ratio which is the total damping (inherent damping plus supplementary VD), *t* is time, *ϕ* is phase shift and *a* and *b* are the constant parameters.

Now it is needed to take the first derivative of displacement regarding time to find the velocity. Therefore, the velocity can be arranged as:

$$\dot{x}(t)=-A\omega_{f}sin(\omega_{f}t+\phi )+e^{-\xi \omega_{n}t}(a\omega_{d}cos\omega_{d}t-b\omega_{d}sin\omega_{d}t)-\xi \omega_{n}e^{-\xi \omega_{n}t}(asin\omega_{d}t+bcos\omega_{d}t)$$ (7)

t2≤t≤t4

By taking into account the below initial conditions, applying them to the velocity equation and making some new assumptions, four equations with four unknowns will be obtained. Solving four equations simultaneously leads to the final results. The procedure could be described as:

$$x(t_{2})={+\Delta }_{0},\;x(t_{4})=-\Delta_{0},\;\dot{x}(t_{2})=0,\;\dot{x}(t_{4})=0,$$ (8)

Then knowing that normally *ξ*≪1, then *ξ*^2^≅0. So, the following equations can be achieved:

$$\omega_{n}=\omega_{d},\;\beta =\beta_{d}\to \frac{\omega_{f}}{\omega_{n}}=\frac{\omega_{f}}{\omega_{d}},\;e^{+\xi \omega_{n}(t_{3}-t_{2})}≅e^{-\xi \omega_{n}(t_{2}-t_{1})}$$ (9)

Now by introducing the foregoing boundary conditions to Eqs (4) & (7), taking *π*_1_ = *π*/*β*, and substituting *t*_4_ = *t*_2_+*π*/*ω*_*f*_ in Eq (8), the below equations arise:

$$x(t_{2})=Acos(\omega_{f}t_{2}+\phi )+B+e^{-\xi \omega_{n}t_{2}}(asin\omega_{n}t_{2}+bcos\omega_{n}t_{2})={+\Delta }_{0}$$ (10)

$$x(t_{4})=-Acos(\omega_{f}t_{2}+\phi )+B+e^{-\xi \omega_{n}t_{2}}(asin(\omega_{n}t_{2}+\pi_{1})+bcos(\omega_{n}t_{2}+\pi_{1}))={-\Delta }_{0}$$ (11)

$$\dot{x}(t_{2})=-A\omega_{f}sin(\omega_{f}t_{2}+\phi )+e^{-\xi \omega_{n}t_{2}}(a\omega_{n}cos\omega_{n}t_{2}-b\omega_{n}sin\omega_{n}t_{2})-\xi \omega_{n}e^{-\xi \omega_{n}t_{2}}(asin\omega_{n}t_{2}+bcos\omega_{n}t_{2})=0$$ (12)

$$\dot{x}(t_{4})=A\omega_{f}sin(\omega_{f}t_{2}+\phi )+e^{-\xi \omega_{n}t_{2}}(a\omega_{n}cos(\omega_{n}t_{2}+\pi_{1})-b\omega_{n}sin(\omega_{n}t_{2}+\pi_{1}))-\xi \omega_{n}e^{-\xi \omega_{n}t_{2}}(asin(\omega_{n}t_{2}+\pi_{1})+bcos(\omega_{n}t_{2}+\pi_{1}))=0$$ (13)

Considering the above-mentioned equations and solving them together, results in finding the basic response parameters of the proposed hybrid SDOF system.

### 3. Finding the maximum displacement and its time lag for the structures equipped with HDM

To proceed with design for dynamic loads, it is needed to formulate MD of the single-degree-of-freedom system equipped with the new hybrid damper. As mentioned earlier, the equations are established for the lower branch of Fig 2.

Rearranging Eqs (10) and (11) can result in the following statements:

$$Acos(\omega_{f}t_{2}+\phi )={+\Delta }_{0}-B-e^{-\xi \omega_{n}t_{2}}(asin\omega_{n}t_{2}+bcos\omega_{n}t_{2})$$ (14)

$$Acos(\omega_{f}t_{2}+\phi )={+\Delta }_{0}+B+e^{-\xi \omega_{n}t_{2}}(asin(\omega_{n}t_{2}+\pi_{1})+bcos(\omega_{n}t_{2}+\pi_{1}))$$ (15)

and confronting Eqs (14) and (15) by using the trigonometric rules will result in an important equation that contributes a lot to getting to the final results:

$$e^{-\xi \omega_{n}t_{2}}\left(asin(\omega_{n}t_{2}+\frac{\pi_{1}}{2})cos(\frac{\pi_{1}}{2})+bcos(\omega_{n}t_{2}+\frac{\pi_{1}}{2})cos(\frac{\pi_{1}}{2})\right)=-B$$ (16)

or in another form:

$$e^{-\xi \omega_{n}t_{2}}=\frac{-B}{\left(asin(\omega_{n}t_{2}+\frac{\pi_{1}}{2})cos(\frac{\pi_{1}}{2})+bcos(\omega_{n}t_{2}+\frac{\pi_{1}}{2})cos(\frac{\pi_{1}}{2})\right)}$$ (17)

By following the same steps for Eqs (12) and (13) the below equations are reachable:

$$A\beta sin(\omega_{f}t_{2}+\phi )=e^{-\xi \omega_{n}t_{2}}(acos\omega_{n}t_{2}-bsin\omega_{n}t_{2})-\xi e^{-\xi \omega_{n}t_{2}}(asin\omega_{n}t_{2}+bcos\omega_{n}t_{2})$$ (18)

and simplifying Eq (18) leads to:

$$A\beta sin(\omega_{f}t_{2}+\phi )=e^{-\xi \omega_{n}t_{2}}((a-\xi b)cos\omega_{n}t_{2}-(\xi a+b)sin\omega_{n}t_{2})$$ (19)

Again, at this moment the Eq (13) is recalled. As it is apparent by changing the sides in this equation the below relation is obtained:

$$A\beta sin(\omega_{f}t_{2}+\phi )=-e^{-\xi \omega_{n}t_{2}}(acos(\omega_{n}t_{2}+\pi_{1})-bsin(\omega_{n}t_{2}+\pi_{1}))+\xi e^{-\xi \omega_{n}t_{2}}(asin(\omega_{n}t_{2}+\pi_{1})+bcos(\omega_{n}t_{2}+\pi_{1}))$$ (20)

and reshaping Eq (20) transforms it to:

$$A\beta sin(\omega_{f}t_{2}+\phi )=-e^{-\xi \omega_{n}t_{2}}(\left(a-\xi b)cos(\omega_{n}t_{2}+\pi_{1})-(\xi a+b)sin(\omega_{n}t_{2}+\pi_{1}\right))$$ (21)

At this time employing Eqs (19) and (21) simultaneously yields to:

$$\frac{acos(\omega_{n}t_{2}+\frac{\pi_{1}}{2})-bsin(\omega_{n}t_{2}+\frac{\pi_{1}}{2})}{asin(\omega_{n}t_{2}+\frac{\pi_{1}}{2})+bcos(\omega_{n}t_{2}+\frac{\pi_{1}}{2})}=\xi$$ (22)

or

$$tan\left(\omega_{n}t_{2}+\frac{\pi_{1}}{2}\right)=\frac{a-\xi b}{\xi a+b}$$ (23)

Referring to the Eqs (14) and (15) and doing the summation of sides reveals that:

$$Acos{(\omega_{f}t_{2}+\phi )={+\Delta }_{0}+e}^{-\xi \omega_{n}t_{2}}\left(acos(\omega_{n}t_{2}+\frac{\pi_{1}}{2})sin(\frac{\pi_{1}}{2})-bsin(\omega_{n}t_{2}+\frac{\pi_{1}}{2})sin(\frac{\pi_{1}}{2})\right)$$ (24)

or in a simplified form, it can be stated as:

$$\frac{Acos(\omega_{f}t_{2}+\phi )}{sin(\frac{\pi_{1}}{2})}=\frac{{+\Delta }_{0}}{sin(\frac{\pi_{1}}{2})}{+e}^{-\xi \omega_{n}t_{2}}\left(acos(\omega_{n}t_{2}+\frac{\pi_{1}}{2})-bsin(\omega_{n}t_{2}+\frac{\pi_{1}}{2})\right)$$ (25)

Introducing Eqs (17) and (22) to Eq (25) and doing mathematical simplifications yields the following statement which is revealed to be the fundamental equation to derive the subsequent formulas for the hybrid SDOF system.

$$\frac{Acos(\omega_{f}t_{2}+\phi )}{sin(\frac{\pi_{1}}{2})}=\frac{{+\Delta }_{0}}{sin(\frac{\pi_{1}}{2})}-\frac{\xi B}{cos(\frac{\pi_{1}}{2})}$$ (26)

or

$$Acos(\omega_{f}t_{2}+\phi )={+\Delta }_{0}-\frac{\xi Bsin\pi_{1}}{1+cos\pi_{1}}$$ (27)

On the other hand, by adding Eq (19) to Eq (21) it can be obtained that:

$$\frac{A\beta sin(\omega_{f}t_{2}+\phi )}{\left(\xi a+b)sin(\frac{\pi_{1}}{2}\right)}={+e}^{-\xi \omega_{n}t_{2}}\left(tan(\omega_{n}t_{2}+\frac{\pi_{1}}{2})sin(\omega_{n}t_{2}+\frac{\pi_{1}}{2})+cos(\omega_{n}t_{2}+\frac{\pi_{1}}{2})\right)$$ (28)

Now by using Eq (23) and replacing it into Eq (28) it is observed:

$$cos(\omega_{n}t_{2}+\frac{\pi_{1}}{2})=\frac{e^{-\xi \omega_{n}t_{2}}(\xi a+b)sin(\frac{\pi_{1}}{2})}{A\beta sin(\omega_{f}t_{2}+\phi )}$$ (29)

$$sin(\omega_{n}t_{2}+\frac{\pi_{1}}{2})=\frac{e^{-\xi \omega_{n}t_{2}}(a-\xi b)sin(\frac{\pi_{1}}{2})}{A\beta sin(\omega_{f}t_{2}+\phi )}$$ (30)

To further the process some trigonometric rules are required. As a basic rule it is known:

$${sin}^{2}(\omega_{n}t_{2}+\frac{\pi_{1}}{2})+{cos}^{2}(\omega_{n}t_{2}+\frac{\pi_{1}}{2})=1$$ (31)

So, by substituting Eqs (29) and (30) in Eq (31) the following formula is achievable:

$$e^{-\xi \omega_{n}t_{2}}=\frac{A\beta sin(\omega_{f}t_{2}+\phi )}{sin(\frac{\pi_{1}}{2})\sqrt{\left(1+\xi^{2})(a^{2}+b^{2}\right)}}$$ (32)

Then new types of formulas are ensued by replacing Eq (32) in Eqs (29) and (30) as what follows below:

$$cos(\omega_{n}t_{2}+\frac{\pi_{1}}{2})=\frac{\left(\xi a+b\right)}{\sqrt{\left(1+\xi^{2})(a^{2}+b^{2}\right)}}$$ (33)

$$sin(\omega_{n}t_{2}+\frac{\pi_{1}}{2})=\frac{\left(a-\xi b\right)}{\sqrt{\left(1+\xi^{2})(a^{2}+b^{2}\right)}}$$ (34)

It is instructive to know that Eqs (32), (33) and (34) can convert Eq (16) to:

$$A\beta sin(\omega_{f}t_{2}+\phi )=\frac{-(1+\xi^{2})Bsin\pi_{1}}{1+cos\pi_{1}}$$ (35)

By using Eq (35) and replacing it into Eq (32) a new formula is obtained as what is seen in Eq (36). The recently derived equation is an important benchmark to calculate the maximum displacement of the proposed hybrid mechanism (Δ_0_) and its corresponding time (*t*_2_). In the below statement the Eq (36) is represented:

$$e^{-\xi \omega_{n}t_{2}}=\frac{-\sqrt{\left(1+\xi^{2}\right)}B}{cos(\frac{\pi_{1}}{2})\sqrt{\left(a^{2}+b^{2}\right)}}$$ (36)

To calculate the maximum displacement of the hybridized SDOF system (Δ_0_), it is necessary to square both sides of Eqs (27) and (35). Then by doing the summation of the squared equations the maximum displacement of the developed hybrid system can be drawn as what follows:

$$A^{2}={\left(\frac{-(1+\xi^{2})Bsin\pi_{1}}{1+cos\pi_{1}}\right)}^{2}+{\left({+\Delta }_{0}-\frac{\xi Bsin\pi_{1}}{1+cos\pi_{1}}\right)}^{2}$$ (37)

or with rearranging Eq (37) in the other way, it can be explained as:

Δ0=A2−[−(1+ξ2)Bsinπ1β(1+cosπ1)]2+[ξBsinπ11+cosπ1] (38)

By dividing the Eq (38) to *B* and *A* which are the displacement due to the Coulomb friction force and the dynamic displacement of the hybrid SDOF system respectively and with the aid of the Eq (5) two new dimensionless terms are introduced below:

Δ0B=α2(1−β2)2+(2ξβ)2−[−(1+ξ2)sinπ1β(1+cosπ1)]2+[ξsinπ11+cosπ1] (39)

Δ0A=1−(1−β2)2+(2ξβ)2α2[−(1+ξ2)sinπ1β(1+cosπ1)]2+(1−β2)2+(2ξβ)2α[ξsinπ11+cosπ1] (40)

Also, from the Eqs (27) and (35) the time corresponding to maximum displacement (Δ_0_), can be described as:

$$t_{2}=-\frac{\phi }{\omega_{f}}+\frac{1}{\omega_{f}}arccos(\frac{{+\Delta }_{0}}{A}-\frac{\xi Bsin\pi_{1}}{A(1+cos\pi_{1})})$$ (41)

or

$$t_{2}=\frac{\pi }{{2\omega }_{f}}-\frac{\phi }{\omega_{f}}+\frac{1}{\omega_{f}}arccos\left[\frac{-(1+\xi^{2})Bsin\pi_{1}}{A\beta (1+cos\pi_{1})}\right]$$ (42)

As seen above, the maximum displacement of the hybrid SDOF system described in Eq (38) is transformed to two dimensionless forms referred to as Dynamic Amplification Factor (DAF) for SDOF equipped with hybrid damper under external loading as expressed in Eqs (39) and (40). From these mentioned equations, it is seen that the ratios of maximum displacement of the proposed hybridized system (Δ_0_) to the displacement caused by the Coulomb friction and to the dynamic displacement of the SDOF system merely with VD, will change with variation in dimensionless parameters such as *α*, *β* and *ξ*.

Actually, the main aim was to focus on parameters that are effective on dynamic amplification factor. In conventional TMD system, damping ratio and frequency ratio are the most effective ones. However, when it comes to the developed HDM, beside the pre-mentioned parameters, the Coulomb friction plays an important role in fluctuating of dynamic amplification factor. The effectiveness of the Coulomb friction varies by change of mass and any change in the mass of system will result new amounts of friction (resistant) force and subsequently force amplitude ratio. Thus, damping ratio, frequency ratio, mass of structure (needed to find friction force and force amplitude ratio respectively) are the considered parameters to evaluate the behaviour of new developed damping system.

**Fig 3:**
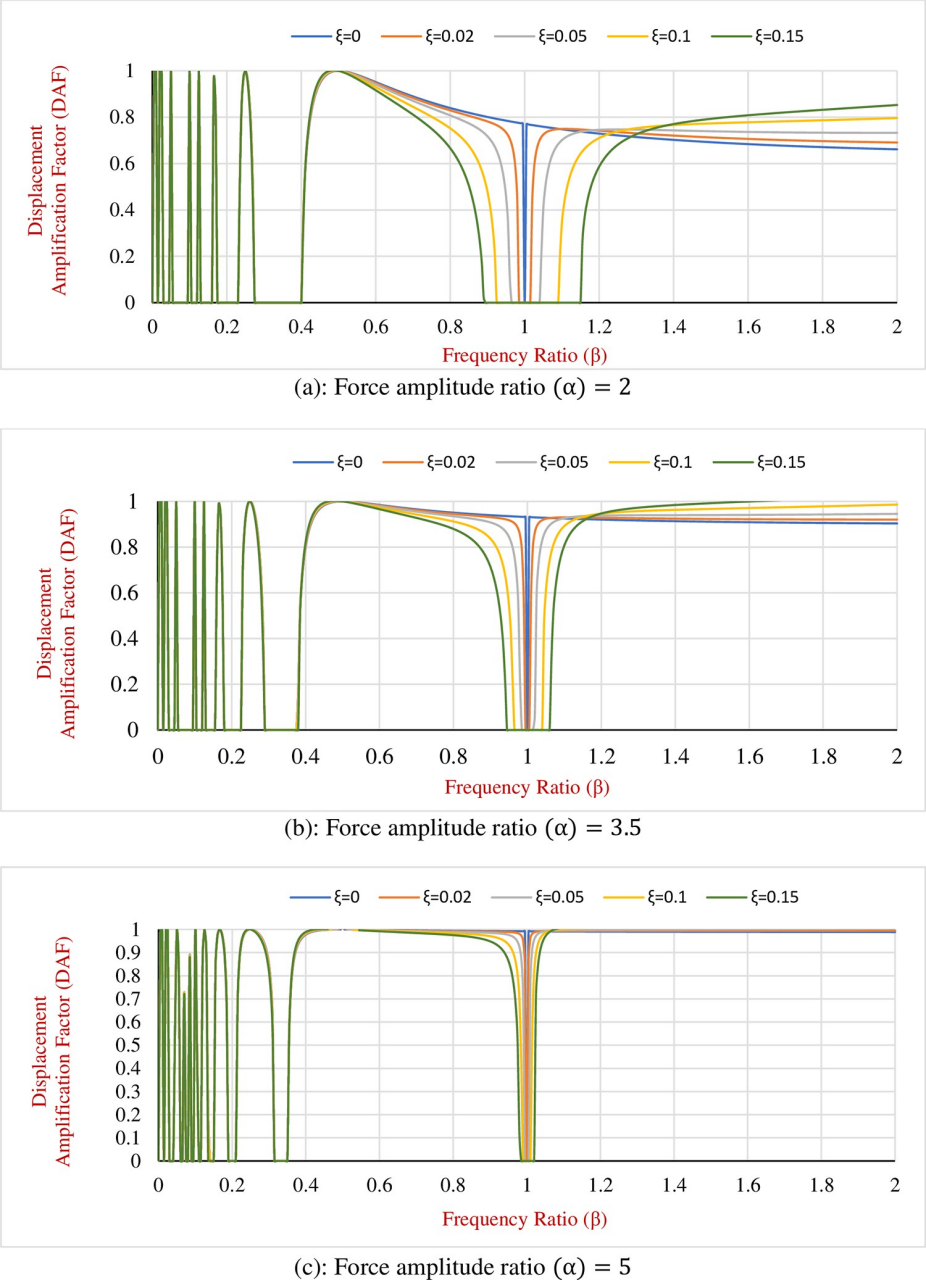
(Δ_0_/*A*) for the hybrid SDOF system subjected to harmonic load for various amounts of *α*, *β* and *ξ*. **DAF** (a): Force amplitude ratio (α) = 2. (b): Force amplitude ratio (α) = 3.5. (c): Force amplitude ratio (α) = 5.

Accordingly, as an illustration of the change in the displacement amplification factor (Δ_0_/*A*) regarding *α*, *β* and *ξ* a particular numerical example is applied. By taking the mass of the SDOF structure (*m*) 50,000 *kg*, the Coulomb friction force (*f*_*d*_) = 15,000 *N* and external force-frequency (*ω*_*f*_) = 2*π* (*rad*/*s*), the DAF graphs for the SDOF fortified with HDM for various amounts of *α*, *β* and *ξ* are represented in Fig 3. As it is observed in the graphs, variation of DAF for SDOF with new hybrid damping mechanism is classified into three main ranges of frequency ratio (*β*) as what is explained below:

### i. Frequency ratio 0<(*β*)<0.5

As is perceived from Fig 3, for this *β* range the combined effect of *ξ* and *α* on the reduction of the Displacement Amplification Factor (DAF) is not tangible, however, this effect is more observable and rises after *β* = 0.5. On the other hand, in this range of frequency ratios, it can be seen that the curves related to different hybrid damping ratios have a horizontal intersection with the zero DAF line.

It is interpreted that the SDOF dynamic system equipped with the HDM confronts consecutive sticking phases during some particular frequency ratio intervals. It is evident from the graphs as the force amplitude ratio (*α*) decreases the length of the sticking intervals increases, this means that by reducing the force amplitude ratio the Coulomb friction becomes more effective in comparison to external lateral load and tends to control the system. However, the sticking phase is not favourable for structural designers and therefore this range of frequency ratio is not considered in dynamic designs.

### ii. Frequency ratio 0.5<(*β*)< inflection point

By inflection point, it is meant that the graphs change their trends. Normally for the hybridized SDOF, this turning point occurs after *β* = 1. In this particular example, for the *α* equal to two the inflection happens at the frequency ratio of 1.3, however, for the *α*’s of 3.5 and 5, this point is shifted back to the frequency ratios of 1.15 and 1.05 respectively. Therefore, it is concluded that the *α* performs a significant role in changing the position of the inflection point.

The tangible range to evaluate the effect of force amplitude ratio is in the range of 2 to 5. For less than 2 the amount of friction force gets closer to amplitude of the external load and there is the possibility that system confronts sticking phase. Also, for ratios greater than 5 the effect of friction force is negligible, and the developed HDM behaves as non-frictional system. Therefore, for better illustrations of plots the range of 2 to 5 is chosen.

By considering the frequency ratios between 0.5 and the inflection point it is evident that both *ξ* and *α* have a noticeable effect on reduction of the Displacement Amplification Factor (DAF). It is comprehended that increasing the *ξ* and *α* results in the higher displacement responses due to external loading and consequently for a constant Coulomb friction force DAF increases as well. Thus, in this case, the behaviour of the SDOF system subjected to harmonic loads and with the Coulomb friction resembles the SDOF systems without friction and therefore DAF is almost 1 in this range of *β*.

However, by magnifying the effect of friction force the effect of the proposed hybris system is more perceivable and the Displacement Amplitude Factor (DAF) decreases subsequently and locates below 1. As the frequency ratio gets closer to 1, the frequency of external load becomes close to the natural frequency of the proposed SDOF and technically the resonance occurs. At this time the minimum *ξ* needed to control the structural response depends on the *α* since as mentioned earlier, reducing the *α* engender the reduction in DAF.

For instance, in this particular example, a 5% damping ratio is required to keep the DAF at 0.6 for the *α* ratio of 2, however, to obtain the same DAF it is necessary to increase the damping ratio to 0.1 and 0.15 for force amplitude ratios of 3.5 and 5 respectively. Therefore, the use of the proposed HDM is meaningful as the inherent damping ratio is 2% and 5% for the steel and concrete structures respectively and as a consequence, it is not sufficient to curb the structural displacements during the severe excitations.

If the SDOF oscillatory system is not equipped with a supplementary damping system like the proposed one in this research, as the frequency ratio stands near 1 (resonance case) the structure experiences uncontrollable drifts and it contributes to fatigue in structural components and is followed by damage and collapse of the structure. Applying the developed HDM to the SDOF system decreases the maximum displacement between 1% to 98% for different *α* and VDS damping ratios.

### iii. *β*> frequency of the inflection point

Also from Fig 3, it is observed that when it comes to frequency ratios (*β*) greater than the inflection point increasing the damping ratio of the hybrid system (*ξ*) neutralizes the positive effect of the increment in the force amplitude ratio. In another word, increasing the damping ratio beyond the inflection point leads to a larger DAF. Therefore, the inflection point is called the economic design point and during the design procedure, the natural frequency of the main structure can be set such that the frequency ratio becomes equal to the inflection point ratio. Indeed, DAF at the inflection point is still greater than the DAF at resonance but as the real displacement in the structure is lesser than the displacement in the resonance case, it is referred to as the economic design point. It is instructive to remind that the DAF (Δ_0_/*A*) shows the amount of reduction in displacement of hybrid SDOF in comparison to the displacement of the non-hybrid systems.

Now, this is the time to deal with the time lag of the maximum displacement. From Eq (42) it is observable that time lag (*ω*_*f*_*t*_2_) is a function of *ϕ*, *α*, *β* and *ξ*, However, by ignoring the effect of phase shift (*ϕ*) it would be only a function of three dimensionless parameters (*α*, *β* and *ξ*). By multiplying e Eq (42) in *ω*_*f*_ and doing some simplification the following statement is obtained:

$$\omega_{f}t_{2}=\frac{\pi }{2}-\phi +arccos\left[\frac{-(1+\xi^{2})Bsin\pi_{1}}{A\beta (1+cos\pi_{1})}\right]=\frac{\pi }{2}-\phi +f(\alpha ,\beta ,\xi )$$ (43)

In which,

$$f(\alpha ,\beta ,\xi )=arccos\left[-\frac{\sqrt{{\left(1-\beta^{2}\right)}^{2}+{\left(2\xi \beta \right)}^{2}}}{\alpha }\frac{(1+\xi^{2})sin\pi_{1}}{\beta (1+cos\pi_{1})}\right]$$ (44)

**Fig 4:**
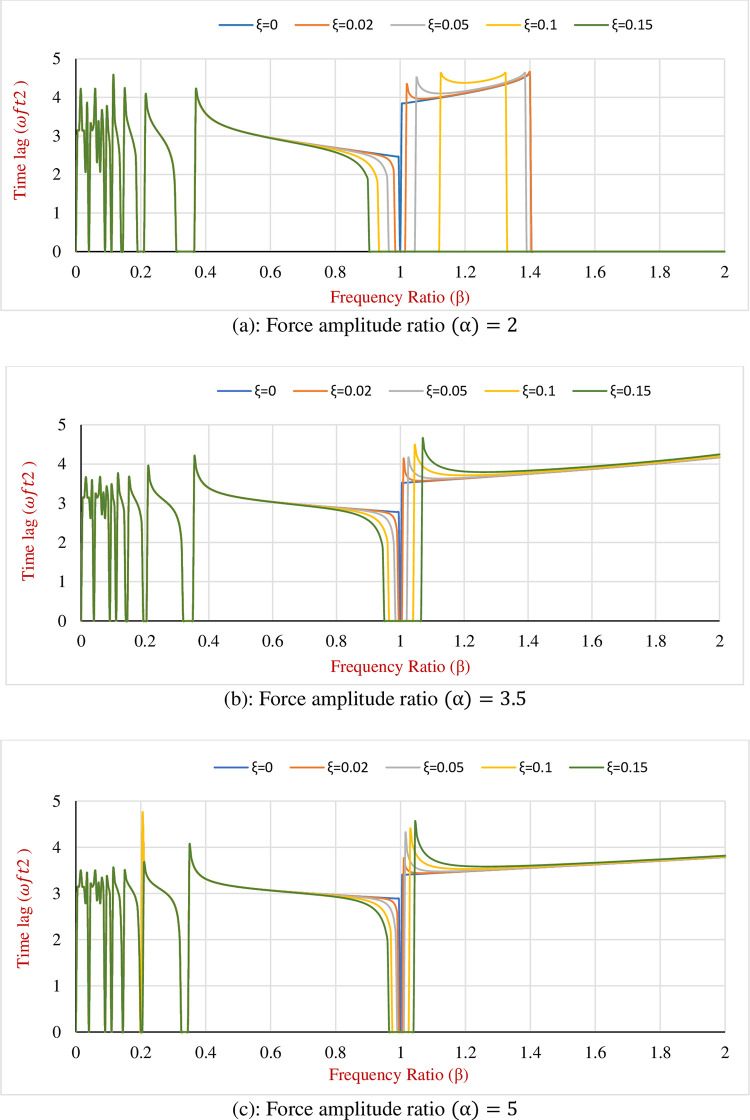
Time lag of the maximum displacement (*ω*_*f*_*t*_2_) for the proposed hybrid SDOF system for various amounts of t *α*, *β* and *ξ*. (a): Force amplitude ratio (α) = 2. (b): Force amplitude ratio (α) = 3.5. (c): Force amplitude ratio (α) = 5.

By assuming the numerical example introduced earlier the variation of time lag for the hybrid SDOF system regarding various amounts of frequency ratio (*β*), damping ratio (*ξ*) and force amplitude ratio (*α*) can be seen as what is illustrated in Fig 4.

From Fig 4 and its illustrated graphs the following findings can be derived:

1. Utilizing the Hybrid Damping Mechanism (HDM) contributes to decreasing the time lag of the Maximum Displacement (MD) for the frequency ratios in the range of 0.5 to 1. It can be interpreted that due to the increase in the Coulomb friction force the velocity of the hybridized SDOF decreases. On the other hand, the maximum displacement decreases as well, yet the rate of decrease in the maximum displacement is lesser in comparison to the velocity decrement. Therefore, the time lag shows a descending trend. In this particular example installing the proposed HDM on an SDOF system resulted in a decrement in time lag in the range of 0 to 36%.
2. Implementing the proposed HDM on the SDOF dynamic system increases the time lag for the frequency ratios greater than 1. Although by applying the HDM the maximum displacement decreases, the velocity decreases at a lesser rate and consequently time lag rises. In the previous example, the amount of increment is between 0 to 24%.
3. A higher damping ratio (*ξ*) results in a lower or greater time lag depending on the frequency ratio, however, the effect of the damping ratio is more sensible for *β* between 0.85 to 1.15.
4. Without any change in the amplitude of the external force as the Coulomb friction rises, the force amplitude ratio decreases and therefore the friction controls the hybrid SDOF system. As a consequence, the length of the sticking phases in the graphs (horizontal intersection of graphs with frequency ratio axis) increases with the increase in the friction force.
5. For all amounts of the *α*, the time lag locates between 0 to 4.64. It implies that the total range of the time lag is the same for various *α*’s, however, by decreasing *α* the SDOF system confronts more sticking intervals.
6. The minimum time lag occurs at frequency ratios close to resonance (*β* = 1). At this stage, the mass reaches its maximum energy, then it leads to experience of the maximum velocity as well. Therefore, the time lag corresponding to the maximum displacement is minimum at the resonance stage since the mass reaches to the peak velocity.
7. In the sticking phase as the hybrid SDOF system stops its movement, neither the maximum displacement nor the time lag can be defined and therefore there is not any amount on the graphs that can be attributed to them.
8. For all damping sudden sharp changes has been observed at *β* = 0.2. However, in these graphs since the colour of damping ratio equal to 0.1 is dominant, it seems that it only happens for *ξ* = 0.1. In fact, for frequency ratios between 0 to 0.4, there are some fluctuations in amplification factor because system is confronted with stick and release conditions frequently, due to presence of Coulomb friction. This sudden increment is also a result of experiencing the sticky phase and the subsequent release state.

### 4. Obtaining the coefficients of the steady-state response of the hybrid system (*a* and *b*)

In this section, the two coefficients for the steady-state response of the hybrid SDOF system are derived. Then by finding the coefficients, a complete steady-state solution for the SDOF system equipped with HDM can be presented, however, to obtain the mentioned coefficients some mathematical calculations are needed.

Replacing Eq (27) into Eqs (14) and (15), results in Eqs (45) and (46) with two unknowns. By solving the Eqs (45) and (46) for two unknowns (*a* and *b*), the values of *a* and *b* can be determined respectively. The procedure is explained as:

$$e^{-\xi \omega_{n}t_{2}}(asin\omega_{n}t_{2}+bcos\omega_{n}t_{2})=\xi Btan\frac{\pi_{1}}{2}-B$$ (45)

$$e^{-\xi \omega_{n}t_{2}}(asin(\omega_{n}t_{2}+\pi_{1})+bcos(\omega_{n}t_{2}+\pi_{1}))=-\xi Btan\frac{\pi_{1}}{2}-B$$ (46)

From Eq (45) *b* is calculated and expressed versus *a*:

$$b=\frac{e^{+\xi \omega_{n}t_{2}}(\xi Btan\frac{\pi_{1}}{2}-B)-asin\omega_{n}t_{2}}{cos\omega_{n}t_{2}}$$ (47)

and introducing Eq (47) into Eq (46) reveals the value of *a* as:

$$a=e^{+\xi \omega_{n}t_{2}}\left(\frac{-Bsin(\omega_{n}t_{2}+\pi_{1})}{cos\frac{\pi_{1}}{2}}-\frac{\xi Bcos(\omega_{n}t_{2}+\pi_{1})}{cos\frac{\pi_{1}}{2}}\right)$$ (48)

From Eq (48) it is evident that *a* is a function of the below parameters:

a=f(ξ,α,β,ϕ,B) (49)

Repeating the same steps for *b* leads to the following statement:

$$b=e^{+\xi \omega_{n}t_{2}}\left(\frac{-Bcos(\omega_{n}t_{2}+\pi_{1})}{cos\frac{\pi_{1}}{2}}+\frac{\xi Bsin(\omega_{n}t_{2}+\pi_{1})}{cos\frac{\pi_{1}}{2}}\right)$$ (50)

Again, it is seen that *b* is also a function of the five parameters:

b=f(ξ,α,β,ϕ,B) (51)

To verify the effect of *ξ*, *α* and *β* on the coefficients of the steady-state response of the hybrid system, it is assumed that *ϕ* = 0 and also *B* is a constant value.

Therefore, by considering the aforesaid assumptions the Eqs (49) and (51) reduce to:

a=f(ξ,α,β) (52)

b=f(ξ,α,β) (53)

In what follows the variation of the response coefficients (*a* and *b*) with the change in the amounts of damping ratio, force amplitude ratio and frequency ratio (*ξ*, *α* and *β*) is illustrated.

**Fig 5:**
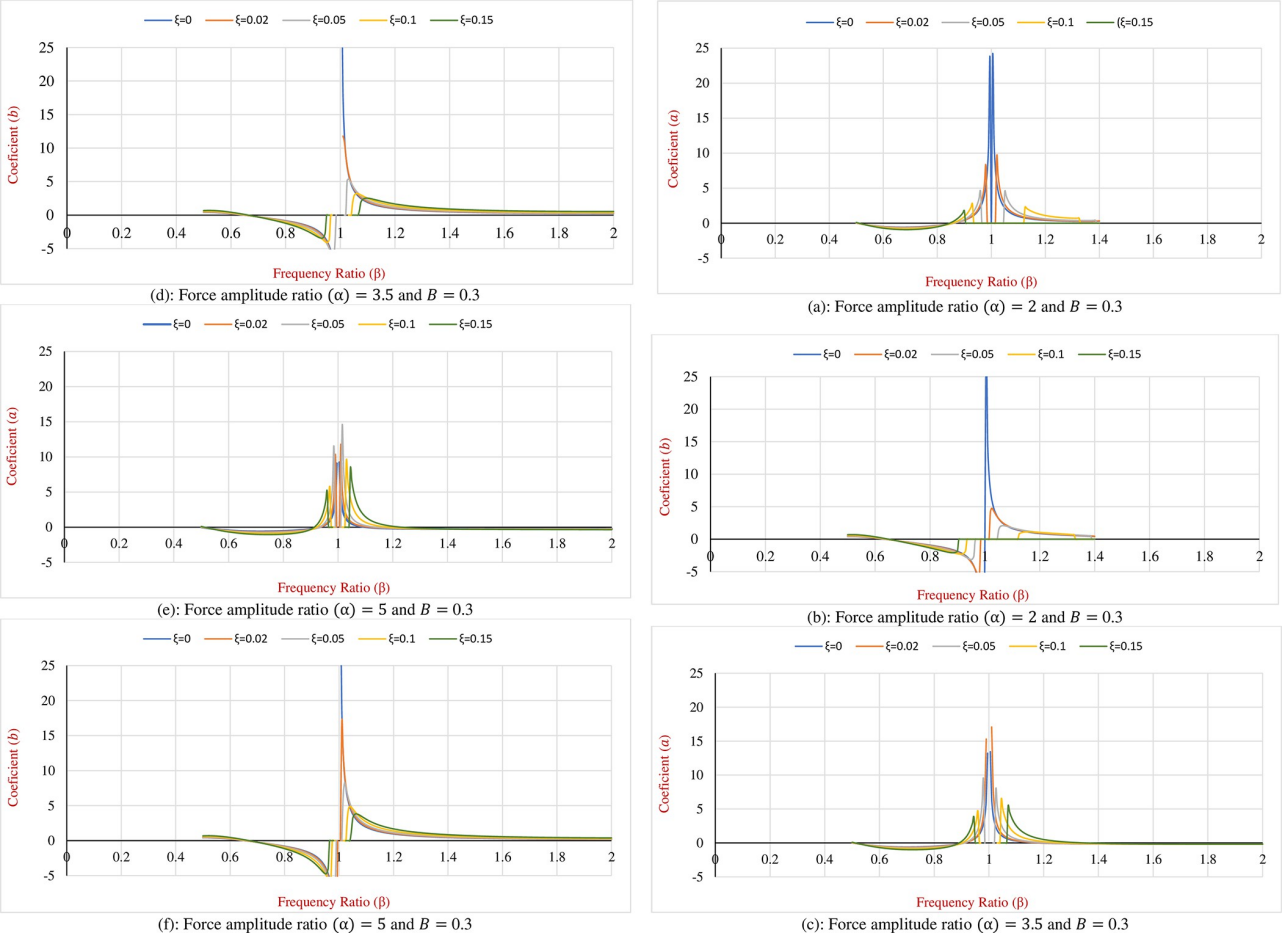
Variation of the coefficients of the steady-state response of the proposed hybrid SDOF system for various amounts of *α*, *β* and *ξ*. (a): Force amplitude ratio (α) = 2 and *B* = 0.3. (b): Force amplitude ratio (α) = 2 and *B* = 0.3. (c): Force amplitude ratio (α) = 3.5 and *B* = 0.3. (d): Force amplitude ratio (α) = 3.5 and *B* = 0.3. (e): Force amplitude ratio (α) = 5 and *B* = 0.3. (f): Force amplitude ratio (α) = 5 and *B* = 0.3.

As is seen in Fig 5 the following conclusions regarding the coefficient’s graphs can be drawn:

- As the amplitude ratio of force increases (*α*) the range of *β* in which the possible amounts for the steady-state response coefficients (*a* and *b*) can be found increases as well. In this particular example, the minimum range belongs to *α* of 2 which is located between *β* of 0.5 to 1.4.
- As it is evident there is not any amount that can be allocated to *a* and *b* for frequency ratios

lesser than 0.5. This is attributed to the sticking phase that occurs in this range of frequency, therefore the SDOF hybrid system stops oscillating and there is not any steady-state response definable for the system

- Increasing the amplitude ratio of force due to a decrease in the Coulomb friction results in a lower magnitude of the coefficient *a*, however, meanwhile, the coefficient *b* has an ascending trend and reaches 36 and 37 for force amplitude ratios of 3.5 and 5 respectively.
- The effect of the damping ratio (*ξ*) on the variation of the response coefficients (*a* and *b*) is more tangible for the frequency ratios in the range of 0.85 to 1.15.

### 5. Determining the minimum required force amplitude ratio to avoid the getting stuck in the SDOF systems equipped with HDM

During the derivation of the formulas for the steady-state response of the hybrid SDOF systems, the basic assumption is that the oscillatory motion has zero-duration stick phases. It means to imply that the back-and-forth motion will not trap in the sticking phase. To, fulfil this condition it is needed to set the amplitude of the external load in a way that the mass constantly oscillates in the sliding phase. Therefore, it is obvious that the following equation must be satisfied:

$$|P_{0}sin\omega_{f}t-kx(t)|\geq f_{d}$$ (54)

From the oscillation trend, it is known that the most critical condition occurs at time intervals *t*_2_ and *t*_4_, respectively. At these points, the system velocity becomes zero and if the externally applied force is less than the restoring force inserted by the spring, the stick phase will happen. So, recasting Eq (68) for time *t*_2_ leads to:

$$|P_{0}sin\omega_{f}t_{2}-kx(t_{2})|\geq f_{d}$$ (55)

Knowing that *x*(*t*_2_) = Δ_0_ and by introducing Eqs (38) and (42) to the Eq (55) the following statement can be shaped:

$$-\left(\sqrt{{\left(\frac{A}{B}\right)}^{2}-{\left[\frac{-(1+\xi^{2})sin\pi_{1}}{\beta (1+cos\pi_{1})}\right]}^{2}}+[\frac{\xi sin\pi_{1}}{1+cos\pi_{1}}]\right)+\alpha sin\left(\frac{\pi }{2}-\phi +arccos[\frac{-(1+\xi^{2})Bsin\pi_{1}}{A\beta (1+cos\pi_{1})}]\right)=1$$ (56)

Then the Eq (56) is simplified and the boundary line for *α* will be gained. This boundary line will assist structural engineers to avoid the sticking in their calculations and is used as a handful guideline in dynamic analysis of the SDOF systems reinforced with HDM.

By assuming a particular case in which *ϕ* = 0, a simpler form of Eq (56) can be extracted as:

$$-\left(\sqrt{{\left(\frac{A}{B}\right)}^{2}-{\left[\frac{-(1+\xi^{2})sin\pi_{1}}{\beta (1+cos\pi_{1})}\right]}^{2}}+[\frac{\xi sin\pi_{1}}{1+cos\pi_{1}}]\right)+\alpha \left[\frac{-(1+\xi^{2})Bsin\pi_{1}}{A\beta (1+cos\pi_{1})}\right]=1$$ (57)

or

$${\left(\frac{A}{B}\right)}^{2}=\left[{\left(1+((1+\xi^{2})\sqrt{{\left(\frac{1}{\beta }-\beta \right)}^{2}+{\left(2\xi \right)}^{2}}+\xi )\frac{sin\pi_{1}}{1+cos\pi_{1}}\right)}^{2}+{\left[\frac{(1+\xi^{2})sin\pi_{1}}{\beta (1+cos\pi_{1})}\right]}^{2}\right]$$ (58)

and by replacing *A* and *B* from Eq (5), the force amplitude ratio boundary can be defined as:

$$\alpha \geq \sqrt{\left[{\left(1-\beta^{2}\right)}^{2}+{\left(2\xi \beta \right)}^{2}][{\left(1+((1+\xi^{2})\sqrt{{\left(\frac{1}{\beta }-\beta \right)}^{2}+{\left(2\xi \right)}^{2}}+\xi )\frac{\sin\pi_{1}}{1+\cos\pi_{1}}\right)}^{2}+{\left[\frac{(1+\xi^{2})\sin\pi_{1}}{\beta (1+\cos\pi_{1})}\right]}^{2}\right]}$$ (59)

**Fig 6:**
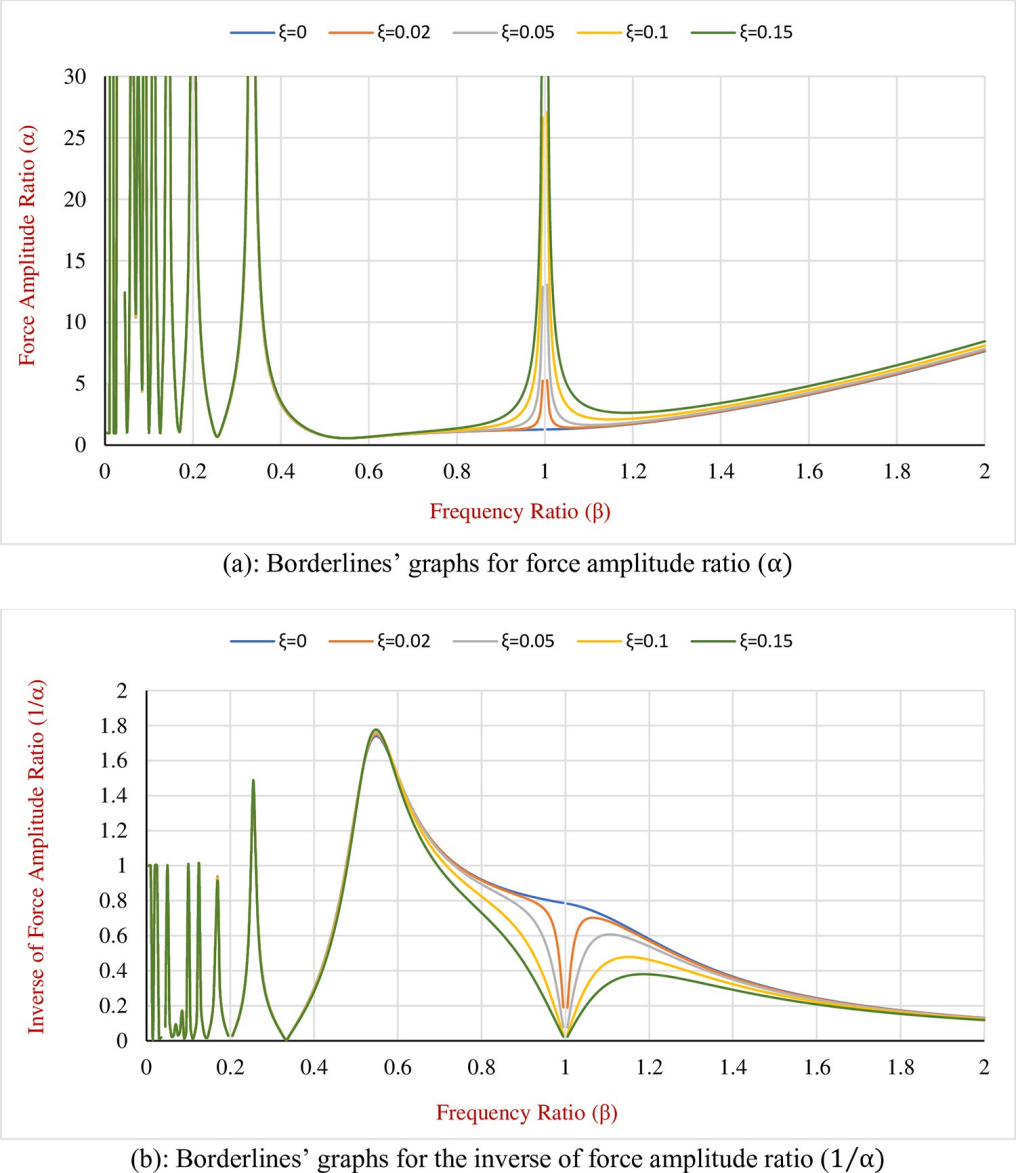
Boundary lines for α and its inverse (1/α) versus β. (a): Borderlines’ graphs for force amplitude ratio (α). (b): Borderlines’ graphs for the inverse of force amplitude ratio (1/α).

Then Eq (59) is solved for different frequency and damping ratios (*β* and *ξ*) and the results are illustrated by the graphs as shown in Fig 6.

As is seen in Fig 6 by ignoring the fluctuations of curves in the frequency range of 0 to 0.5 and separating the rapid changes that occur at resonance (β = 1) the overall trend of α regarding the variation of *β* is linear. Knowing that the amounts of force amplitude ratio for frequency ratios of 0.5 and 2 are 0.6 and 8.4 respectively, the linear relation can be described as what follows:

α=5.2×β−2 (60)

Therefore, the Eq (59) is converted to a simple for as represented in Eq (60). By choosing the frequency ratio and consequently the force amplitude ratio (α) greater than the amounts obtained from Eq (60) the designing procedure of the structure will be arranged in a way that the vibrating system will not experience any sticking phase. For force amplitude ratios lesser than amounts of Fig 6(a) the hybridized SDOF system faces sticking intervals. Even at the resonance if some viscous damping ratio is added to the system the external loading will not be enough to neutralize the Coulomb friction and the system stops oscillating and the HDM is not effective anymore, therefore, adding the HDM in the Coulomb systems must be carried out with the full attention to avoid negative consequences.

Also, for 1/α the Eq (61) can be derived. In some cases, using 1/α can be easier as its range is located between 0 to 0.9 in this particular example. By dividing 1 to both sides of Eq (60) it is observed:

$$\frac{1}{\alpha }=\frac{1}{5.2\times \beta -2}$$ (61)

Eqs (60) and (61) are the borderlines of the amplitude ratio of the force and its inverse respectively and eventually, they can be used as guidelines for structural designers during the designing process. The illustrated curves will aid designers to select the frequency ratio such that the minimum displacement and the non-sticking condition are satisfied simultaneously. It is useful to remind that applying the Hybrid Damping Mechanism (HDM) to the SDOF systems must be done carefully to have the best efficiency and the least sticking intervals at the same time.

## 6. Conclusion

In this research, a hybrid damping mechanism for structures is introduced. To evaluate the dynamic behaviour of the proposed Hybrid Damping Mechanism (HDM) under the external harmonic loading, the effect of the VDS was implemented in the principal equation of the motion and formulations for structural response were developed for the SDOF system as a basic and simple structure.

By solving the equation of motion for boundary conditions the main response parameters such as the Maximum Displacement (MD) and its corresponding time lag (the radian elapsed by the structural mass to reach the maximum displacement) were formulated accordingly. Besides the aforesaid parameters, coefficients of steady-state response were calculated as well. A numerical example was applied to the derived formulations of MD and its time lag. The analysis indicated that employing the HDM in the SDOF system results in decreasing the MD in the range of 1% to 98% for various force amplitude and hybrid damping ratios.

In case of *β* between 0 to 0.5, the effects of *ξ* and *α* on reducing the MD or the Displacement Amplification Factor (DAF) are not tangible, however, these effects are more observable at *β*≥0.5. Also, the SDOF faces several non-zero duration sticking phases and therefore the application of HDM must be done carefully at this frequency range.

Considering the frequency ratios between 0.5 and the inflection point (the point at which the DAF graphs change their trends) it is concluded that both *α* and *ξ* have a significant impact on diminishing the DAF. On the other hand, in the pre-illustrated numerical example, increasing *α* shifts the position of the inflection point toward *β* = 1.

In a frequency ratio greater than the inflection point increasing damping has a reverse influence on the DAF and increases it. Thus, the inflection point is the optimal design point and is referred to as the economic design frequency ratio.

Changing *ξ* results in a lower or greater time lag depending on the tuning frequency ratio. The impact of *ξ* in varying the time lag and the steady-state response coefficients (*a* and *b*) is more observable for the frequency ratios between 0.85 to 1.15.

Also, the boundary line was calculated for amplitude ratio of the force to avoid sticking. The exact formulation is somehow complicated; however, it can be approximated by a linear polynomial. The derived formulas and the corresponding graphs can be utilised as guidelines for structural designers during the dynamic designing procedure.

The developed hybrid SDOF system in this carried out research can be considered as a more efficient mechanism alternative to the conventional system for the Tuned Mass Damper (TMD) with high capability to decrease the amplitude of the motion for the auxiliary mass which is the main drawback of the TMDs.

Applying the developed damping mechanism in this study contributes to higher reduction of amplitude of motion through the function of the viscous damper to dissipate vibration beside the action of the Coulomb friction to generate resistant force against the movement. Since the performance of viscous damper enhances by applied friction, therefore action of the proposed hybrid mechanism is noticeable specially in high velocity excitations, where the conventional system may experience excessive motions.

And finally, the proposed HDM mechanism and derived formulations can be expanded to the Multi-Degree-of-Freedom (MDOF) systems to implement in more structural applications. In order to extend the SDOF system to MDOF, since the mass corresponding to each floor is connected to the other floors using spring and damping components, therefore the equation of motion also is expanded to the MDOF by employing new terms. Accordingly, the mass of each floor will have a new maximum displacement and its corresponding time lag. Therefore, more unknown parameters appear in the equation of motion, and it is a little bit more complicated to solve multi-uncoupled equations to find required parameters in comparison to SDOF system.

## References

[1] Bowden F. P., & Leben L. (1939). The nature of sliding and the analysis of friction. *Proceedings of* the Royal Society of London.i Series A. Mathematical and Physical Sciences, 169(938), 371–391.

[2] Ibrahim R. A. (1994). Friction-induced vibration, chatter, squeal, and chaos—part II: dynamics and modelling.

[3] Feeny B., Guran A. S., Hinrichs N., & Popp K. (1998). A historical review on dry friction and stick-slip phenomena, 321–341.

[4] Riaz R. D., Malik U. J., Shah M. U., Usman M., & Najam F. A. (2023, April). Enhancing Seismic Resilience of Existing Reinforced Concrete Building Using Non-Linear Viscous Dampers: A Comparative Study. In *Actuators* (Vol. 12, No. 4, p. 175). MDPI.

[5] Hong H. K., & Liu C. S. (2000). Coulomb friction oscillator: modelling and responses to harmonic loads and base excitations. *Journal of Sound and Vibration*, 229(5), 1171–1192.

[6] Shah M. U., Shah S. W., Farooq S. H., Usman M., & Ullah F. (2023). Experimental investigation of tuned liquid column ball damper’s position on vibration control of structure using different fluids. *Innovative Infrastructure Solutions*, 8(3), 111.

[7] Shah M. U., Usman M., Farooq S. H., & Rizwan M. (2023). Spring-controlled modified tuned liquid column ball damper for vibration mitigation of structures. *Journal of Sound and Vibration*, 545, 117443.

[8] Shah M. U., & Usman M. (2022). An experimental study of tuned liquid column damper controlled multi-degree of freedom structure subject to harmonic and seismic excitations. *Plos one*, 17(6), e0269910. doi: 10.1371/journal.pone.0269910 35696409 PMC9191723

[9] Mostaghel N., Hejazi M., & Tanbakuchi J. (1983). Response of sliding structures to harmonic support motion. *Earthquake engineering & structural dynamics*, 11(3), 355–366.

[10] Parnes R. (1984). Response of an oscillator to a ground motion with Coulomb friction slippage. *Journal of sound and vibration*, 94(4), 469–482.

[11] Younis C. J., & Tadjbakhsh I. G. (1984). Response of sliding rigid structure to base excitation. *Journal of engineering mechanics*, 110(3), 417–432. doi: 10.1061/(ASCE)0733-9399(1984)110:3(417)

[12] Hartog J. D. (1931). Forced vibrations with combined Coulomb and friction. *Transaction of the American Society of Mechanical Engineering*, 53, 107–115.

[13] Makris N., & Constantinou M. C. (1991). Analysis of Motion Resisted by Friction. I. Constant Coulomb and Linear/Coulomb Friction*. *Journal of Structural Mechanics*, 19(4), 477–500.

[14] Hundal M. S. (1979). Response of a base excited system with Coulomb and friction. *Journal of Sound and Vibration*, 64(3), 371–378.

[15] Chen L. Y., Chen J. T., Chen C. H., & Hong H. K. (1994). Free vibration of an SDOF system with hysteretic damping. *Mechanics research communications*, 21(6), 599–604.

[16] Hong H. K., & Liu C. S. (2001). Non-sticking oscillation formulae for Coulomb friction under harmonic loading. *Journal of Sound and Vibration*, 244(5), 883–898.

[17] Riddoch D. J., Cicirello A., & Hills D. A. (2020). Response of a mass-spring system subject to Coulomb damping and harmonic base excitation. *International Journal of Solids and Structures*, 193, 527–534.

[18] Marino L., & Cicirello A. (2021). Multi-degree-of-freedom systems with a Coulomb friction contact: analytical boundaries of motion regimes. *Nonlinear Dynamics*, 104(1), 35–63.

[19] Ziaee M. & Hejazi F. (2022). Development of Non-sticking steady-state solution for structures with hybrid damping mechanism. *Journal of Structures*, 47, 233–245.

[20] Hinrichsen P. F., & Larnder C. I. (2018). Combined and dry friction damping of oscillatory motion. *American Journal of Physics*, 86(8), 577–584.

[21] Araz O., & Kahya V. (2021). Design of series tuned mass dampers for seismic control of structures using simulated annealing algorithm. *Archive of Applied Mechanics*, 91, 4343–4359.

[22] Araz O. (2022). Optimization of three-element tuned mass damper based on minimization of the acceleration transfer function for seismically excited structures. *Journal of the Brazilian Society of Mechanical Sciences and Engineering*, 44(10), 459.

[23] Araz O. (2022). Optimization of tuned mass damper inerter for a high-rise building considering soil-structure interaction. *Archive of Applied Mechanics*, 92(10), 2951–2971.

[24] Araz O., & Kahya V. (2022, May). Optimization of multiple tuned mass dampers for a two-span continuous railway bridge via differential evolution algorithm. In *Structures* (Vol. 39, pp. 29–38). Elsevier.

[25] Araz O., Cakir T., Ozturk K. F., & Kaya D. (2023). Effect of foundation embedment ratio in suppressing seismic-induced vibrations using optimum tuned mass damper. *Soil Dynamics and Earthquake Engineering*, 171, 107981.

[26] Araz O., Elias S., & Kablan F. (2023). Seismic-induced vibration control of a multi-story building with double tuned mass dampers considering soil-structure interaction. *Soil Dynamics and Earthquake Engineering*, 166, 107765.

[27] Araz O., & Farsangi E. N. (2023, June). Optimum tuned tandem mass dampers for suppressing seismic-induced vibrations considering soil-structure interaction. In *Structures* (Vol. 52, pp. 1146–1159). Elsevier.

[28] Araz O. (2020). Effect of detuning conditions on the performance of non-traditional tuned mass dampers under external excitation. *Archive of Applied Mechanics*, 90(3), 523–532.

